# Identifying Key Moments in Type 2 Diabetes Management: A Qualitative Study of the Experiences of People With Type 2 Diabetes and Diabetes Health Coaches

**DOI:** 10.1111/hex.70108

**Published:** 2024-11-21

**Authors:** Jack B. Joyce, Carolyn Newbert, Nicola Guess, Kate Fryer, Caroline A. Mitchell, Liliia Bespala, Elizabeth Morris, Paul Aveyard, Susan A. Jebb, Charlotte Albury

**Affiliations:** ^1^ Nuffield Department of Primary Care Health Sciences University of Oxford Oxford UK; ^2^ Patient and Public Involvement Member of the Study Team; ^3^ School of Medicine and Population Health, University of Sheffield Sheffield UK; ^4^ Faculty of Medicine and Health Sciences, Keele University Keele UK

**Keywords:** co‐research, participatory research, qualitative, remission, thematic analysis, type 2 diabetes

## Abstract

**Objective:**

For people with type 2 diabetes who are overweight, weight loss increases the likelihood of achieving diabetes remission. The aim here was to draw on the experiences of people living with type 2 diabetes and coaches who deliver type 2 diabetes prevention and remission programmes. This was done to develop a service that increases the proportion of people who achieve remission by identifying an effective weight management service.

**Research Design and Methods:**

A qualitative researcher and co‐researcher with type 2 diabetes conducted 37 narrative interviews with adults with type 2 diabetes (October 2022–June 2023) and 16 semi‐structured interviews with health coaches delivering type 2 diabetes programmes in England. Data were analysed using Reflexive Thematic Analysis. Participants were diverse in ethnicity, socioeconomic status, age, gender and years since diabetes diagnosis.

**Results:**

Four themes were generated relating to moments in a person's diabetes care: (1) coming to terms with diagnosis, (2) lightbulb moments, (3) sustaining change as normal and (4) becoming expert/building confidence. These four themes were united under a high‐level interpretivist theme: ‘Same journey, different experience’, capturing the mismatch between a linear rigid care pathway described by coaches and the diversity of experience of people living with type 2 diabetes.

**Conclusions:**

Coaches and people with type 2 diabetes are aligned on their reports of key moments in adapting to diabetes. Participants’ desire for flexibility in their care contrasted with coach reports of rigid service provision. These insights may enable more people with type 2 diabetes to engage and adhere to weight management services aimed at diabetes remission.

## 1 Introduction

Weight loss is the primary driver of remission from type 2 diabetes for people also living with overweight and obesity. (Type 2 diabetes remission is when blood sugar levels return to non‐diabetes level long term [HbA1c below 48 mmol/mol for 3+ months] without using medication.) [1]. Bariatric surgery is the most effective option for achieving significant weight loss and therefore diabetes remission [2], but it is expensive, and the great number of people living with type 2 diabetes and overweight and obesity means it is impossible to offer to most people, and patients are more likely to experience complications than a dietary intervention [2, 3, 4]. A total diet replacement programme (TDR) is the most effective dietary intervention known to achieve remission, achieving an average weight loss of 10% in people taking up this programme, and almost half of these people achieve remission at 1 year [3]. This treatment is offered to patients with type 2 diabetes and overweight in England through the NHS ‘Path to Remission’ programme. Whilst TDR interventions have been shown to be effective for those who accept it, uptake in routine care is low and people can find adherence challenging [3]. Those finding TDR unacceptable or unsuitable could benefit from alternative weight loss interventions that may enable them to lose sufficient weight to achieve remission.

People from minoritised ethnic groups, those experiencing deprivation, disability and people with learning disabilities are more likely to develop type 2 diabetes and experience complications earlier [5, 6]. Women are also more likely to experience complications from type 2 diabetes [7]. Research reveals how these groups often experience harmful intersectional stigma [8, 9], including blame, judgement and differential treatment [10], which can cause distress, poorer quality interactions with professionals and adversely affect their health and health care [11, 12]. These groups are most in need, and most likely to benefit from remission programmes, but are underrepresented in the development of current interventions [13], and underserved by their implementation adding to the low adherence and sustainability of these interventions in the long term. Effective care services for people with type 2 diabetes should incorporate the needs and wishes of all [14]. Patients respond well to interventions when services are delivered in ways sensitive to their preferences and experiences [15]. To develop treatment approaches that are acceptable, suitable and more likely to be successful, particularly for those at higher risk from type 2 diabetes and underserved by research, it must be understood why current approaches may not be most optimal.

Studies exploring success in managing diabetes often relate it to how well a patient adheres to treatment [16, 17] and whether they achieve a desired clinical outcome [1]. This is complicated by many different components that influence weight management over time, including biological factors, family and social culture, and the availability and accessibility of food options [18]. Tying success and failure to an individual's actions, and not the treatment approach or wider context may therefore be problematic. Perceptions of patient success/failure can influence the uptake and adherence of treatment approaches and a more useful approach could be ‘experimenting with change’ [19]. Understanding how individuals conceive of success/failure in living with type 2 diabetes may help develop more supportive treatment approaches.

We sought to understand the experiences of a diverse group of people with type 2 diabetes and health coaches delivering diabetes prevention and remission interventions to inform the development of an enhanced remission service. We hypothesised that improving the range, choice and presentation of weight loss programmes could increase uptake and persistence with weight loss programmes and therefore improve remission rates. This may be more acceptable, suitable and more likely to achieve longer term success, particularly for those for whom existing remission interventions are unsuccessful or unwanted.

## 2 Methods

This study was part of a programme of work to develop and test a new service for remission with the aim of increasing the number of people newly diagnosed with type 2 diabetes who take up and adhere to weight management programmes aiming to achieve diabetes remission. We reasoned that understanding the experiences of people with type 2 diabetes and coaches delivering existing diabetes prevention and remission interventions would allow us to understand whether existing provisions met people's needs or could be adapted. Reporting follows the Consolidated Criteria for Reporting Qualitative Studies (COREQ) [20].

### 2.1 Participatory Approach

To place the experience of people with type 2 diabetes centrally in this research, participatory research methods were used [21]. People with type 2 diabetes were involved throughout, including the design, data collection and analysis, in the form of a diverse patient and public involvement panel recruited via the Deep End Research Alliance (DERA) and the Weight Management patient advisory group at Oxford and involvement of a co‐researcher with lived experience of type 2 diabetes. (DERA is a group of 12 individuals from a variety of ethnic minority and underserved communities across the South Yorkshire and Humber regions.) 'Co‐research' is research done *with* or *by* the public rather than *about* or *for* them [22]. Lived experience co‐researcher involvement aims to demystify, democratise, challenge traditional hierarchies and improve the overall quality of research [23] by including their voices [24]. A co‐researcher (C.N.) offered reflections throughout the research complementing our academic reflections, producing richer results more relevant to people with type 2 diabetes. She undertook an academic qualitative interviewing course and was asked for her reflections after each interview.

### 2.2 Recruitment of Participants

Adults with type 2 diabetes (patient‐participants) were recruited across England. We developed a sampling frame with members of a patient advisory group to guide recruitment for maximum variation in experience living with type 2 diabetes (Supporting Information S1: 1 and 2). We aimed for a purposive sample with demographic diversity. Sampling was iterative and responsive to our reflections developed from the data. As patterns in the data were observed, further sampling based on those observations was done. Demographic information, including age, gender, ethnicity, diabetes duration, current role, living arrangements, dietary limitations, any disabilities and index of multiple deprivation (IMD) decile was collected. This was to ensure maximum variation and diversity of sample was achieved. Activities to recruit included leafleting/speaking at community events and community and religious centres, via word‐of‐mouth, through newsletters and email lists, on social media and radio interviews. Translation support for people who could not understand English and additional support for people with learning disabilities was offered. All participants were reimbursed with shopping vouchers and carers at their hourly rate of pay.

Coaches were recruited from a private healthcare provider delivering diabetes prevention and remission services. Services (face‐to‐face, digital or remote) are offered on the NHS via referral from a GP or via self‐referral with an eligible blood test as part of NHS England's ‘Healthier You NHS Diabetes Prevention Programme’ [25]. Coaches were recruited opportunistically and via snowballing with support from a senior coach. They were reimbursed at their hourly rate of pay.

Recruitment for both participant sets ceased when the population reached information power [26] that data collected was varied, relevant and held sufficient detail and insights to address the study aim. This was assessed throughout the collection process.

### 2.3 Ethical Considerations

All participants gave informed consent before participation and re‐confirmed their consent post‐interview. Study information was available via participant information sheets, a video and in translated formats (including EasyRead) provided at the point of arranging an interview. Ethical approval from the University of Oxford was granted (reference number: R81647/RE001).

### 2.4 Data Collection

Narrative interviews with patient‐participants were conducted with participants asked a single question ‘Can you tell me about the effect of type two diabetes on your life, starting from when you were first diagnosed?’. This question was co‐developed with the lived experience co‐researcher and reviewed by the patient advisory group. The aim was to understand the emotional impact of interventions; perceptions of ‘success’ and ‘failure’; contextual factors that may have facilitated or hindered referral acceptance and perseverance; how concepts and perceptions changed over time; as well as other factors patients presented as important in their narratives. Narrative interviews allow for participants to share and emphasise aspects of their experience they perceive as important to them, focussing on participant‐led (rather than researcher‐led) data collection. This was followed by prompting questions, if necessary, to elicit further details (Supporting Information S1: 3). Semi‐structured interviews with coaches were conducted by J.B.J. and focused on views on existing diabetes prevention and remission interventions and how they are experienced by people with type 2 diabetes while remaining open to topics the coaches revealed as important (Supporting Information S1: 4).

One qualitative researcher (J.B.J.) and lived experience co‐researcher (C.N.) conducted and audio‐recorded the patient‐participants interviews between October 2022 and June 2023. They had no personal or professional relationship with the participants. The qualitative researcher was present for all interviews and the co‐researcher with lived experience for seven patient‐participant interviews (Supporting Information S1: 2). The interview guides were developed within the research team with input from a patient advisory group. Interviews lasted up to 36 min (median 22 min). Interviews were conducted by telephone for the accessibility and comfort of participants. Field notes were taken by J.B.J. during each interview, which included reflexivity notes, suggested iterations to recruitment and assessed the strength of the dialogue. Data collection and analysis were grounded in interpretivism. The data were co‐created by researchers and participants, and then interpreted by the researchers during analysis.

### 2.5 Data Analysis

Audio recordings of interviews were anonymised and transcribed verbatim. The data were analysed by J.B.J. using reflexive inductive thematic analysis to identify ‘particular patterns of shared meaning across the data set’ [27]. This was supplemented with the constant comparative approach [28] to interrogate (in)consistencies in views across interviewees. Data retrieval and analysis were facilitated by NVivo 1.6. Analysis followed five phases [1]: Data familiarisation, J.B.J. read and reread the transcripts while noting initial thoughts in a reflexivity journal [2]. Coding the transcripts were inductively coded with descriptive codes to closely represent what the participants reported—this was repeated iteratively to ensure codes captured all features (see Table 1 for more details) [3]. Theme development, related codes were collated into larger units of potential meaning [4]. Theme review, themes were discussed, checked and refined in relation to the data set [5]. Defining and name, themes were defined and sense‐checked with all authors.

**Table 1:** Techniques used for maintaining reflexivity throughout reflexive thematic analysis (RTA).

Phase of RTA	What we did	Why/How we did it
Familiarisation of data	Listen to and re‐read data.	During familiarisation preliminary observations and notes were documented in a reflexivity diary. Noting possible codes, feelings and impressions, points of interest, assumptions about responses and tentative ideas for patterns of meaning. Early thoughts and ideas were shared with a co‐researcher to invite them to reflect.
Descriptive coding of interview transcripts	Describe and document participants’ experiences, perceptions, and narratives as and how they are expressed.	The coding reflected participants’ actual words to be faithful to how they chose to present their narrative. We wanted to represent their voices as transparently as possible and not overshadow participants’ intended meanings with our own interpretations. All data were coded descriptively in Nvivo 1.6. This allows others to see how conclusions were drawn from the data. Definitions and criteria for codes were documented using memos in Nvivo 1.6. Codes were shared and regularly discussed with a second qualitative researcher to actively challenge assumptions.
Generating themes	Generate findings that go beyond description and provide more meaningful insights into a person's experience living with type 2 diabetes.	The process of bringing together codes was iterative allowing for continual reflection so interpretations were responsive to new insights and ways of pulling together patterns of meaning. Notes made during the first two phases were drawn on. A reflexivity diary documented initial thoughts, assumptions and possible clustering of codes. These were discussed with second qualitative researcher and co‐researcher to identify beliefs, assumptions and preconceptions.
Reviewing of themes	Iteratively review themes in relation to the data and each other.	Reflection by other researchers to seek richer and more nuanced understandings of the data. Ensuring that patterns are underpinned by a central concept and that concepts are recognisable to others. An additional high‐level interpretivist theme was generated in discussion with a second qualitative researcher to unite all themes.
Defining and naming themes	Capture the essence of the pattern of meaning each theme represents.	Names of themes continually refined throughout to best capture the pattern of meaning it represents. Names and definitions developed in discussion with a second qualitative researcher and co‐researcher. These were then presented to the wider interdisciplinary team along with extracts to challenge assumptions and understandings and to make sure names and definitions reflect what is in the data. After naming, themes were shared with the patient advisory group to see whether and how they resonated with their experiences.
Reporting findings	Presenting findings in a cogent narrative	Writing was undertaken recursively throughout the analysis. Different document versions retained to view the evolution of the writing. The ordering of themes was considered to build a cogent narrative, with the uniting high‐level theme presented last to bring themes together. Report was shared with members of the research team for feedback and reflections, and findings shared with the patient advisory group to reflect and assess whether it resonated with their experiences.

The qualitative researcher (J.B.J.) conducted phases 1–5 and had reflexive discussions with a second qualitative researcher (C.A.) and co‐researcher (C.N.) to actively challenge assumptions. Data from patient‐participants and coach‐participants were coded separately, but themes generated and reviewed based on both sets. A high‐level theme was developed based on the similarities and differences identified by using the constant comparative approach. The reported themes were presented to three members of the patient advisory group for reflections [29] and ensure the research resonated with their experience. J.B.J. wrote up the findings.

One of the epistemological assumptions of the interpretivist paradigm is that there is not a single reality but many realities that could be articulated based on the standpoints and positions of the author(s). These findings are offered as one possible interpretation of these individuals’ experiences. Findings were regularly discussed with an interdisciplinary team of qualitative researchers, nutritionists, general practitioners, coaches and members of a patient advisory group. This study follows Lincoln and Guba's [30] approach to enhance trustworthiness and rigour of qualitative research studies. Table 1 details the techniques used to maintain reflexivity throughout the six phases of reflexive thematic analysis (RTA) [27].

## 3 Results

Fifty‐three people were interviewed (37 patient‐participants; 16 coaches) (detailed information, Table 1). Over half of participants (21/37) identified as non‐White British. Around half of participants reported a disability–including four people with learning disabilities. Some patient‐participants reported experiencing a diabetes prevention programme and coach‐delivered weight loss support. All coaches were trained to deliver the NHS National Diabetes Prevention Plan (via face‐to‐face groups and digital) with some also delivering remission services. Training to deliver these services did not include behaviour change techniques (e.g., motivational interviewing) but some, typically more experienced coaches, reported an awareness of behaviour change approaches. Coaches reported gender and ethnicity only. There were more male than female coaches (11 and 5, respectively), and they predominantly identified as White British and South Asian (13 and 3). (FT = full time, PT = part time, IMD = Index of Multiple Deprivation decile. IMD data taken via postcodes and https://imd-by-postcode.opendatacommunities.org/imd/2019, 1 = most deprived, 10 = least deprived.)

Four themes were generated focussing on key moments participants highlighted as important and united these under a high‐level interpretivist theme. These were recurrently described as points where perceptions of success and failure became relevant in their journey [1]: coming to terms with diagnosis [2], lightbulb moments [3], sustaining change and [4] becoming expert/building confidence. Although the way key moments were navigated varied, these four critical moments themselves were consistent across the diverse population, and so are reported together. Individuals are referred to using anonymised identifiers. Quotes illustrate and exemplify findings (Tables 2, 3, 4, 5, 6).

**Table 2:** Patient‐participant detailed demographic information.

Participant ID	Years since diagnosis	Age	Gender	Ethnicity	Role	Disability	Living arrangement	Dietary limitation?	IIMD
ND1_01	5–10	76	Female	British Pakistani	FT	Did not say	2	No	7
ND1_02	10+	70	Male	British Pakistani	Retired	No	2	Yes	4
ND1_03	10+	68	Male	White British	Retired	No	2	No	9
ND1_04	2–5	63	Female	White British	Retired	Yes	1	No	1
ND1_05	0–2	30	Female	British Pakistani	Unemployed	No	1	Yes	1
ND1_06	10+	52	Female	British Pakistani	Other	Yes	2‐5	Yes	2
ND1_07	5–10	61	Male	South Asian	Other	No	2	No	9
ND1_08	0–2	72	Female	White British	Retired	Yes	1	No	1
ND1_09	0–2	38	Female	South Asian	FT	No	1	No	1
ND1_10	5–10	72	Male	White British	Retired	No	2	No	3
ND1_11	10+	55	Female	British Pakistani	PT	No	2‐5	No	2
ND1_12	10+	68	Female	African Caribbean	Retired	Yes	2	Yes	8
ND1_13	10+	69	Male	White British	Retired	Yes	1	Yes	3
ND1_14	5–10	71	Male	African Caribbean	FT	Did not say	2	No	4
ND1_15	2–5	62	Male	White British	Retired	No	2	No	8
ND1_16	10+	80	Male	White British	Retired	No	2	No	6
ND1_17	10+	61	Male	White British	PT	Yes	2	No	6
ND1_18	0–2	35	Female	British Pakistani	PT	No	2‐5	Yes	2
ND1_19	10+	53	Female	Sikh Indian	PT	Yes	1	No	1
ND1_20	0–2	36	Female	British Pakistani	FT	No	2‐5	No	2
ND1_21	5–10	24	Female	South Asian	PT	Yes	2‐5	Yes	4
ND1_22	2–5	28	Male	South Asian	PT	No	1	Yes	3
ND1_23	0–2	58	Female	White British	FT	Did not say	2	Yes	5
ND1_24	10+	63	Female	White British	FT	No	2	Yes	1
ND1_25	2–5	57	Female	White British	PT	No	2‐5	No	8
ND1_26	5–10	73	Female	African Caribbean	PT	Yes	2	No	7
ND1_27	5–10	65	Male	Sikh Indian	Other	Did not say	1	Yes	4
ND1_28	10+	63	Male	White British	Unemployed	Yes	2	No	10
ND1_29	10+	54	Female	White British	Other	Yes	2	No	8
ND1_30	2–5	63	Female	White British	Other	Yes	5+	No	10
ND1_31	2–5	69	Male	White British	Other	Yes	5+	No	9
ND1_32	10+	55	Female	British Pakistani	PT	Yes	2	Yes	7
ND1_33	0–2	48	Female	Sikh Indian	Retired	Yes	2‐5	No	6
ND1_34	5–10	49	Female	White British	FT	Did not say	1	Yes	9
ND1_35	0–2	53	Male	British Pakistani	FT	Yes	2	Yes	2
ND1_36	0–2	44	Male	Sikh Indian	PT	Yes	2	No	3
ND1_37	0–2	46	Male	South Asian	Other	No	2‐5	Yes	1

**Table 3:** Theme 1. Illustrative quotes.

Quote number	Participant ID	Quote
1	ND1_02	But that first week, it was just feeling sorry for myself. Why me God? What did I do wrong? Is this the rest of my life? Has everything come to an end now? I went through all of those emotions.
2	ND1_21	When I found out, I think it was a big kind of shock to my system. Both my parents were diabetics as well. And I think just seeing how they thought, it just kind of…I kind of felt like my kind of, like…my life ended before it has started. And I think there was a lot of shame attached to as well, for a lot of shame during that time. I think, umm, I don't know what it is…I think it was just…I kind of realized that this is all my fault.
3	ND1_05	It was…I wasn't really expecting. I was expecting according to my family history. I wasn't expecting it to me getting it. It did take a while for me to get my head around it. in the beginning.
4	ND1_10	when I had…when I was diagnosed with diabetes, I was shocked because I'm a middle‐class bourgeois bloke, and I didn't think I'd get it.
5	ND1_19	it's been progressive, the effects of it, in terms of having to accept. First of all, it took me a number of years to accept the diabetes mentally
6	HC1_02	they are…are sometimes a little bit angry, sometimes a little bit confused, when we do speak to them in that first session.
7	HC1_08	I think a large reason why that is you have very demotivated individuals. And for a doctor or a nurse just to say ‘when you leave this practice, could you please think about signing up for an obesity clinic or a diabetes clinic or smoking clinic’, you know, these people are given the free choice. They're going to forget or can't be bothered shortly afterwards for whatever reason. They're psychologically motivated, or very demotivated.

**Table 4:** Theme 2. Illustrative quotes.

Quote number	Participant ID	Quote
8	HC1_14	That shifting mindset was really key because that then made them realize that, yeah, I do need to go to the gym or I do need to go for an hour's walk or half an hour's walk or whatever it is they can do, to help them become fitter, healthier and happier as I'd call it.
9	HC1_07	And it's just about what session is going to create that light bulb moment for them? Is it the introductory session of most common reasons for being pre diabetic? Or is it potentially session four, or five, which is in a few months’ time where we talk about carbohydrate intake?
10	ND1_02	Not fully understanding insulin, not fully understanding diabetes at that time, I was going into hypos left, right and centre. A decision had to be made that I needed to change my career.
11	ND1_03	So, when I did have it, umm, I read about it…that you could reverse it by just, you know, changing, you know, your diet and exercise. So, I started exercising, one hour a day walking, changing my food, you know, things like carbs that, you know, it was more prone to having diabetes or having, you know, high diabetes.
12	ND1_10	I was able to live my lifestyle as I wanted. I carried on with the drinking, umm, so beer, mainly, probably around an average of…I'm just trying to think about…15 to 20 times a week. And…but I…when I split up, I could then start to change my diet. […*discussion about reducing calories*…] And actually, in the last year…last two years, I haven't drunk at all, mainly because drinking just made no sense to me. I just gave it up… I didn't and… I didn't like the feeling that alcohol brought in me.
13	ND1_31	I was a very heavy drinker for a long period in my life. Err, and I had to be a my…in the days when the NHS could afford to treat people, I was actually treated by a consultant for fatty liver. And it's that realization that being…being somebody who has a problem with alcohol is not a functioning alcoholic.
14	HC1_06	I think that realisation is quite key. And I think with that you [coaches] need to maybe reflect questions back to them [clients] a little bit more and let them explore solutions and find ways around the barriers that they're facing rather than you giving them all the answers.

**Table 5:** Theme 3. Illustrative quotes.

Quote number	Participant ID	Quote
15	ND1_11	The past few weeks, it's been a bit hectic because we've had some family weddings and some functions and my diet has been like all over the place.
16	ND1_19	I'm constantly changing my diet [chuckles]. I do… I eat carbs. I think when you accept it and you become more responsible, then… and I still struggle. I don't like to use the word diet. So, I dieted all my life. And I just put on double, triple the weight. So, it almost feels like a punishment.
17	HC1_11	And sometimes as well, you will get a lot of participants that, in the nicest way possible, will come up with a lot of excuses. And you need to be as positive as you can to emphasize with them, but push them forward. Regardless of the excuses, we need to get you from A to B.
18	HC1_04	It is not pushing them because pushing is the wrong word, but encouraging them to rethink what they think is normal.
19	ND1_05	I thought I'd be more…In the progress, I thought I'd have lost more weight and I thought I'd have got controlled a bit more than I have at the moment. I'm living just…I'm just living with it.
20	ND1_18	I've always wanted to lose weight or always been a bit heavy then I've lost weight a few stones and I've gone back to it when I got married and had my first child so I gained a lot of weight. And then, err, after him, I tried to eat not unhealthy… tried to eat healthy then lose a little bit away and then I got pregnant again and had my second child. Because of the children obviously, I'm up and down.
21	ND1_20	Basically, it's all with you. I think it's mind… I mean you've got to first prepare yourself, accept that this is the situation. Accept that you have this issue. Accept that these are permanent changes to your life. Once you do that it's plain sailing.
22	ND1_12	I've never been on a diet as to speak of. Umm, it's, umm, I've just always cut down whatever I'm eating I will have a smaller portion to lose the weight and get back to how I was before. And I keep losing weight and keep putting it back on.

**Table 6:** Theme 4. Illustrative quotes.

Quote number	Participant ID	Quote
23	HC1_02	But they see themselves negatively and their motivation just fluctuates, that's very, very normal. And I'm trying to make them understand that it is normal because some of them quite…are quite harsh on themselves
24	HC1_05	Reminding participants of how far they've come, constantly saying you're still with us, you still in the programme, you're still improving, you're still here. Little things like that. So, it's again building a lot of motivation to change how they see themselves.
25	HC1_07	They need to understand the psychology behind the fact that this is their personal journey. And it doesn't… It doesn't concern anyone else because anyone else isn't going to help them. It is only them, and it's on them and when they realise that they'll become more confident.
26	ND1_02	I've talked to various experts, attending online and face to face seminars and so on, I feel I've gained quite a lot of knowledge which I'm quite happy to share with other people and that's what I try and do.
27	ND1_32	Umm I'm probably the healthiest I've been other than aging. That's the…What am I now, so probably the healthiest I've been in about 35 years.
28	ND1_15	I feel like I've healed myself in a way. And I think, to myself more like credit for it. Like I put a lot of effort into like help…like healing myself emotionally and physically and I don't know…Like, I feel…I feel I think I need to give myself more credit

### 3.1 Themes

#### 3.1.1 Theme 1: Coming to Terms with a Diagnosis of Type 2 Diabetes

Patient‐participants explained that the start of their experience of diabetes was laden with emotion (affect). Most patient‐participants described their immediate post‐diagnosis feelings negatively often with a sense of shame, finality and self‐blame for being diagnosed (Quotes 1 and 2).

Patient‐participants expressed surprise that, despite their prior experience or knowledge of diabetes, they did not see themselves as the type of person who would develop diabetes. Their surprise had ramifications for how quickly they adapted to living with type 2 diabetes. Most often, not seeing themselves as the type of person to develop diabetes impeded their adaptation (Quotes 3–5). On the other hand, people with learning disabilities reported divergent experiences and for them, beginnings were mostly not emotional. This was underpinned by the absence of an explicit diagnosis and explanation from their doctor. Most people with learning disabilities reported that they were given another tablet from the doctor and told to ‘avoid cakes’ (ND1_30) and similar simplistic instructions.

Coaches recognised the period immediately post‐diagnosis could be emotional for their clients. Many coaches related this to their worry that client emotions (such as frustration, uncertainty and shame) might affect how well a client responded to their coaching (Quote 6). Coaches explained that these emotions often led to client demotivation, with one saying, ‘And they just kind of beat themselves up about it and kind of spiral and feel quite demotivated as well’ (HC1_14). Coaches blamed referring clinicians for the demotivation they observed, describing clinician referrals in terms of ‘forcing’ people onto programmes with little information or agency (‘you need to not force anybody to go on to a programme [.] you're not giving them the freedom to make their own decision’, HC1_11). Coaches felt these ‘forced’ referrals were hampering the initial stages of the programme they were delivering (Quote 7).

Two coaches described their strategies to support clients coming to terms with a diagnosis of type 2 diabetes that could enhance engagement with their coaching and the diabetes programme: ‘Because some people can be really nervous when they first start, especially if they are very overweight. And another thing is like it can be more personalised for them rather than a one size fits all situation’ (HC1_14). HC1_04, a South Asian coach, explained their supporting tactic: ‘ve had that a few times happen, especially that I do [speak] other languages, and so I speak to them in a different language’. In general, these approaches were initiated by the coaches who used their own skills to enhance engagement with the intervention.

#### 3.1.2 Theme 2: Lightbulb Moments Throughout the Journey

Both patient and coach‐participants narrated moments of realisation or ‘lightbulb moments’. Both sets of participants described these moments positively, as points at which a person comes to terms with what diabetes means to them. Lightbulb moments were described in two ways: as occurring after some time, that, with hindsight, a positive change had been sustained, and occurring because of a disruptive event in a person's life, usually resulting in a sudden realisation they needed to make a change. These disruptive events were unrelated to diabetes but prompted changes that resulted in positively adjusting to living with diabetes. Events varied from problematic alcohol consumption and a life‐changing accident to relationship breakdown or career change.

Coaches conceptualised these as opportunistic moments they looked to create and capitalise on, with one coach rhetorically saying ‘what session is going to create that lightbulb moment?’ (HC1_07, Quote 9). In contrast, patients did not report ‘lightbulb moments’ as something they realised they were having at the time. They reported that it was only when looking back they realised a lightbulb moment had occurred at a key point. Some coaches described the consequential nature of realisations and the culmination of efforts that led to lightbulb moments for clients (Quote 8).

Every coach reported trying to get these lightbulb moments to happen but stressed the greater chance of positive change occurring if their client had the realisation on their own (‘If they come up with ideas by themselves, they're more in control, and that gives them the power to implement them a little bit more.’ HC1_06). Patient‐participants demarcated life before and after a lightbulb moment. Lightbulb moments featured aspects of learning about themselves, about diabetes and practical matters (e.g., reducing carbohydrate intake) that strongly influenced how they lived with diabetes (Quotes 10 and 11). These moments, after change occurred, were frequently reported alongside other decisions people had made, often involving alcohol consumption (Quotes 12 and 13), and leaving a career or relationship (Quotes 10 and 12). Decisions prompted changes which led to them being more positive about living with diabetes. Coaches reported helping clients to identify a realisation moment as a way of consolidating positive changes in behaviour (Quote 14).

#### 3.1.3 Theme 3: Sustaining Change as a New Normal

Sustaining change was challenging for patient‐participants. Patient and coach‐participants reported different barriers and enablers for sustaining changes in living with diabetes. Participants lamented limitations imposed by diabetes on their everyday lives. They found life events such as birthdays, weddings, funerals, etc. could disrupt changes (Quote 15). Patient‐participants conveyed a sense of responsibility to achieve and sustain weight loss which was followed by self‐blame if/when they believed they were unsuccessful (Quote 16). Coaches expressed mixed views of these scenarios.

Some coaches sought to help individuals sustain changes by asking them to celebrate their ‘new normal’ (HC1_15) in living with diabetes and described that ‘normal’ as something their clients needed to realise to adapt more quickly. Others emphasised needing to push clients as they were ‘creating excuses’ (HC1_13) not to embrace change (Quotes 17 and 18). Pushing clients appeared to be a consequence of delivering a linear programme (Quote 17) that does not account for clients’ desire to make and sustain change, and their overall struggle to realise their ‘new normal’.

Patient‐participants expressed a strong desire to make more progress towards goals. Even those reporting pride in accomplishments (see Theme 4) desired even greater success (Quote 19). Some participants reported continually attempting to make changes were ‘like a punishment’ (Quote 15) and could easily be thwarted by disruptive life events (Quote 20). Most interviewees reported gradually adapting to diabetes. For some, this was described as ‘returning to normal’ (ND1_03), but for most, including coaches, this was adjusting to a ‘new normal’, which was a life with diabetes rather than returning to their life before diabetes. Importantly, embracing change was linked to continued success in managing diabetes (Quote 21), whereas patients desire to return to a pre‐diagnosis ‘normal’ was associated with regaining weight (Quote 22).

#### 3.1.4 Theme 4: Success Within the Journey: Becoming Expert/Building Confidence Means Learning to Live With Diabetes in One's Own Way

Patient‐participants reported success, real or desired, with diabetes as reaching a point of sustaining a consistent treatment approach that works for them. This moment was noted with personal pride–that diabetes had spurred them to make and sustain positive changes (as in Theme 3), but they had gone further and become expert in their diabetes. For most, ‘remission’ was ambiguous ('I'm at a point where I'm off medication for a few months now and I do feel conflicted if I'm a diabetic and this is part of my life. I guess that I would like to find out but like, I think that's remission', ND1_22), and for others, it was never mentioned. Coaches responded to people's negative view of themselves by seeking to instil confidence by normalising feelings, through championing all success, and encouraging self‐reflection (Quotes 23 and 24).

Many coaches reported that people with diabetes had to work to become confident in adapting to diabetes (Quote 25) and most patient‐participants viewed their current life positively, reflecting on the process and work to adapt to diabetes. Patient‐participants felt the outcome of this work meant they had improved their overall health and not just diabetes (Quotes 27 and 28). Participants commonly reported reducing alcohol consumption and becoming more physically active as achievements they had worked towards. Only a couple of participants reported specific successes relating to remission, whereas most reported more generally that they had become healthier and expert in managing their condition. Some patient‐participants had, in adapting to life with diabetes, begun supporting others’ adaptation. This included supporting family, friends, colleagues and for one of the participants, giving time to Diabetes UK (Quote 26). Some participants reported success as not (yet) 'succumbing to insulin' (ND_35). Participants saw insulin use as the 'end of the road' (ND_07). Participant awareness of remission was low and therefore not understood to be an achievement. Other changes like reducing alcohol consumption and increasing physical activity were celebrated by participants. Similarly, becoming an expert in self‐management was highly valued, and avoiding insulin was a significant motivator.

## 4 High‐Level Theme: Same Journey, Different Experience

Descriptive themes are drawn together under a high‐level interpretive theme. Across the diverse patient‐participants, key moments were broadly consistent. It was how these were managed that varied. Age, living arrangements, and ethnicity affected this management. Younger participants had trouble balancing work with diabetes which was less of a concern for older participants who had more flexibility in their lifestyles. Those living alone adapted more quickly versus those with families, and this was compounded by those who came to live alone post‐diagnosis. South Asian participants had different accounts, with changes infrequently sustained. Patient‐participants experienced key moments differently; the time taken to reach, and the order of moments, was influenced by the different participants’ circumstances. This variability and diversity contrasted with the rigid care system described by coaches, where they described leading time‐focussed group sessions where everyone needed to be at the same stage of their journey at the same time to realise the strongest benefit. Whilst they acknowledged everyone's experience was different, they were mostly unable to accommodate this difference in their services, although many attempted this. A mismatch was noticed between a linear rigid support programme described by coaches and the diversity and fluidity of patient journeys.

Participants valued control and choice over their treatment programme. Control was unavailable for participants with learning disabilities who reported no choices and felt no control over diabetes, instead following a highly systematised approach with very little information.

## 5 Discussion

In this interview study of 37 patient‐participants and 16 coaches, people were asked about their experience living with type 2 diabetes and current diabetes prevention and remission services. Four themes were generated and united under a high‐level interpretivist theme based on patient and coach interviews which could be used to inform and improve remission programmes. The time immediately post‐diagnosis had the strongest emotional impact on patient‐participants. Coaches postulated a highly individualised approach, at odds with current group‐based delivery, could be more helpful in motivating change more rapidly. Research suggests specific information provision [31] and support for managing emotional tumult [32], which are continuous and encouraging [33], are predictors of success. Remission services for people newly diagnosed could consider individual support, that group‐based delivery may rival a patient's desire for tailored and continued support especially when coming to terms with diagnosis.

Patients described lightbulb moments where they felt successful and realised they needed to change. Realisations led to, and were the result of, positive changes in behaviour as reported by patients and coaches. Changes in behaviour, and how they were appraised by the individual, often follow realisations [34, 35]. Remission services could encourage patients to reflect on achievements and invite them to interpret reflections positively–such as reframing challenges as opportunities for future success. Sustaining change is viewed by many as unachievable and participants described being stuck in a cycle of weight loss and weight gain. Those who sustained changes strived to improve their overall health. Few participants discussed remission and achieving remission was not a goal for nearly all participants. Current remission services do not necessarily address the societal, family and cultural demands on individuals choosing to pursue remission [36, 37]. Reframing remission to account for the key moments in a patient's journey with type 2 diabetes may mean it becomes meaningful and achievable for those choosing to pursue remission.

Patient‐participants and coaches emphasised challenges around tailored care. Vasconcelos Silva et al. (2022) found a similar pattern in their study on Australian diabetes care–there exists a desire for care suiting individuals, but there is a lack of adequate resources for professionals to support people with diabetes to find and make individualised adaptations. Evidence recognises diabetes care personalised to the individual, with them collaborating in their care, as optimal [14, 38, 39] suggesting remission services offering different approaches for individuals to experiment with could be more advantageous. Research finds patients describe current care as primarily consisting of information provision, whereas what patients want is a collaborative partnership to discuss their own management of diabetes [31, 40]. This research reveals an inflexible system‐centred approach not accommodating the diversity of human experience. A person‐centred approach affording choice and control, and supporting autonomy over received care, may be more widely welcomed.

There is evidence that diabetes remission services have suboptimal uptake and adherence [41] and the data suggest this could occur because they are misaligned with people's experience and expectations. Research evaluating dietary interventions for people from minoritised ethnic groups suggests interventions are less acceptable because they fail to consider sociocultural factors, and do not affirm an individual's autonomy over their diet [42, 43]. Moreover, professionals may further hamper an individual's autonomy by resisting treating people with type 2 diabetes as experts of their diabetes [44]. Remission services may improve outcomes if they are better tailored to peoples’ experience and knowledge. People with diabetes often feel negatively about their state and do not value success. Research shows stigma and perceived responsibility are barriers to feeling successful [45] and that is a barrier to engagement and adherence [34]. Treatment approaches that fail to address stigma are associated with poorer outcomes [12, 46], and in the case of weight loss, increased weight gain in the longer term [47]. Implementing strategies to address stigma, such as creating a more individualised and collaborative care environment, could enhance the effectiveness of remission services. Future research could take a focused approach to examine stigma in this context. Patient‐participants reporting success and achievement recognised and exploited adaptations to diabetes as a broader change in their sense‐of‐self. Shifts in one's identity correlate with positive behaviour change [48]. Remission services could support this by valuing the diverse ways in which patients can succeed. Emphasising the role the person with diabetes has at the centre of their care and how their success could encompass not only clinical remission but also improvements in quality of life may enhance engagement and persistence with a service.

This paper has shown experiences with type 2 diabetes are varied but hinge on key moments. There is a mismatch between a perceived rigidity of care over patients’ desire for flexibility. Treatment approaches affording opportunities for continued support and choice may be more advantageous and including emotional support for newly diagnosed people may help adherence. This qualitative research feeds into the development, delivery and evaluation of a complex service intervention to remit type 2 diabetes. The study demonstrates a commitment to research which is inclusive‐by‐design.

### 5.1 Strengths and Limitations

The strength of this research is the diversity of the sample in relation to participant characteristics and involvement of a patient co‐researcher. This contributes to a fuller understanding of the experience of type 2 diabetes, why remission services may have low uptake and adherence and means our findings are relevant, robust and impactful for more people with type 2 diabetes. Increased attention and efforts to populations underserved and underrepresented, including involving patients in research, can improve health outcomes for all.

A limitation was individuals agreeing to participate may have had an interest in improving interventions for diabetes care or particular views about it that others who are feeling well‐served by the system may not share.

## 6 Conclusion

Coaches and people with type 2 diabetes are generally aligned on reports of significant points in adapting to live with diabetes. Whilst these key moments occurred for most people with type 2 diabetes, they occurred at different times, for different durations, and in different orders. Patient‐participant experiences contrasted with the linear support coaches reported services offered. Adapting to accommodate the different perspectives of people with type 2 diabetes may increase uptake and adherence to remission programmes.

## Author Contributions

**Jack B. Joyce:** conceptualisation, investigation, methodology, formal analysis, project administration, writing–review and editing, writing–original draft, data curation, visualisation. **Carolyn Newbert:** investigation, writing–review and editing, formal analysis, methodology, conceptualisation. **Nicola Guess** and **Liliia Bespala:** writing–review and editing. **Kate Fryer**, **Caroline A. Mitchell**, **Elizabeth Morris**, **Paul Aveyard** and **Susan A. Jebb:** writing–review and editing, funding acquisition. **Charlotte Albury:** investigation, conceptualisation, writing–original draft, writing–review and editing, formal analysis, supervision, project administration, methodology, funding acquisition.

## Patient or Public Contribution

A person with lived experience of type 2 diabetes was involved in the study design, study conduct, analysis and interpretation of the data and preparation of the manuscript. In addition to this, we received feedback from patient advisory group members on aspects of study design. Patient and public contribution follows the NIHR INCLUDE guidelines. More information can be found in our Methods ‘Participatory Approach’ section.

## Conflicts of Interest

In 2022 C.A. was a contracted qualitative methodologist for the Behavioural Insights Team (BIT) for which she was paid personally. She has worked as a consultant qualitative methodologist for Wildfowl Wetlands Trust, Linney Create, and Adelphi Real World, and received personal payment. In 2024 C.A. was an academic advisor to NESTA, and did not receive personal payment. P.A. and S.A.J. were investigators on two publicly funded trials that received total diet replacement treatment from Nestle. Reed Wellbeing and Liva have provided services for health coaching for which they are paid a fee.

## Supporting information

Supporting information.

## Data Availability

The data that support the findings of this study are available in the supplementary material of this article.
