# Supplementary material for: Identifying Key Moments in Type 2 Diabetes Management: A Qualitative Study of the Experiences of People With Type 2 Diabetes and Diabetes Health Coaches
Source: Health Expect. 2024 Nov 21;27(6):e70108. doi: 10.1111/hex.70108 (PMC11581956; doi:10.1111/hex.70108)
Supplement: Supplementary file 1 — Supporting information. [file HEX-27-e70108-s001.docx]

Supplement 1 – Sampling frame

| **Lived experience of type 2 diabetes**   - Years since diagnosis - Age at diagnosis - Age |
| --- |
| **Self-reported gender** |
| **Socio-economic background** |
| **Employment / Role** |
| **Ethnicity** |
| **Disability**   - Mobility |
| **Household Make-up** |
| **Dietary limitations** |
| **Consider themselves overweight** |

Supplement 2 – Participant information

| **Intervie**  **wer** | **Participant** | **Duration (min)** |
| --- | --- | --- |
| JBJ | ND1_01, British Pakistani woman, 76 years old, 5-10 years since diagnosis | 22 |
| JBJ | ND1_02, British Pakistani man, 70 years old, 10+ years since diagnosis | 24 |
| JBJ | ND1_03, White British man, 68 years old, 10+ years since diagnosis | 27 |
| JBJ | ND1_04, White British woman, 63 years old, 2-5 years since diagnosis | 15 |
| JBJ, CN | ND1_05, British Pakistani woman, 30 years old, 0-2 years since diagnosis | 19 |
| JBJ | ND1_06, British Pakistani woman, 52 years old, 10+ years since diagnosis | 20 |
| JBJ | ND1_07, South Asian man, 61 years old, 5-10 years since diagnosis | 31 |
| JBJ, CN | ND1_08, White British woman, 72 years old, 0-2 years since diagnosis | 20 |
| JBJ | ND1_09, South Asian woman, 38 years old, 0-2 years since diagnosis | 30 |
| JBJ, CN | ND1_10, White British man, 72 years old, 5-10 years since diagnosis | 22 |
| JBJ, CN | ND1_11, British Pakistani woman, 55 years old, 10+ years since diagnosis | 31 |
| JBJ | ND1_12, African Caribbean woman, 68 years old, 10+ years since diagnosis | 36 |
| JBJ | ND1_13, White British man, 69 years old, 10+ years since diagnosis | 20 |
| JBJ | ND1_14, African Caribbean man, 71 years old, 5-10 years since diagnosis | 28 |
| JBJ, CN | ND1_15, White British man, 62 years old, 2-5 years since diagnosis | 22 |
| JBJ, CN | ND1_16, White British man, 80 years old, 10+ years since diagnosis | 32 |
| JBJ, CN | ND1_17, White British man, 61 years old, 10+ years since diagnosis | 15 |
| JBJ | ND1_18, British Pakistani woman, 35 years old, 0-2 years since diagnosis | 17 |
| JBJ | ND1_19, Sikh Indian woman, 53 years old, 10+ years since diagnosis | 22 |
| JBJ | ND1_20, British Pakistani woman, 36 years old, 0-2 years since diagnosis | 20 |
| JBJ | ND1_21, South Asian woman, 24 years old, 5-10 years since diagnosis | 25 |
| JBJ | ND1_22, South Asian man, 28 years old, 2-5 years since diagnosis | 29 |
| JBJ | ND1_23, White British woman, 58 years old, 0-2 years since diagnosis | 17 |
| JBJ | ND1_24, White British woman, 63 years old, 10+ years since diagnosis | 20 |
| JBJ | ND1_25, White British woman, 57 years old, 2-5 years since diagnosis | 22 |
| JBJ | ND1_26, African Caribbean woman, 73 years old, 5-10 years since diagnosis | 19 |
| JBJ | ND1_27, Sikh Indian man, 65 years old, 5-10 years since diagnosis | 30 |
| JBJ | ND1_28, White British man, 63 years old, 10+ years since diagnosis | 18 |
| JBJ | ND1_29, White British woman, 54 years old 10+ years since diagnosis | 21 |
| JBJ | ND1_30, White British woman, 63 years old, 2-5 years since diagnosis | 20 |
| JBJ | ND1_31, White British man, 69 years old, 2-5 years since diagnosis | 15 |
| JBJ | ND1_32, British Pakistani woman, 55 years old, 10+ years since diagnosis | 27 |
| JBJ | ND1_33, Sikh Indian woman, 48 years old, 0-2 years since diagnosis | 22 |
| JBJ | ND1_34, White British woman, 49 years old, 5-10 years since diagnosis | 26 |
| JBJ | ND1_35, British Pakistani man, 53 years old, 0-2 years since diagnosis | 31 |
| JBJ | ND1_36, Sikh Indian man, 44 years old, 0-2 years since diagnosis | 24 |
| JBJ | ND1_37, South Asian man, 46 years old, 0-2 years since diagnosis | 29 |
| JBJ | HC1_01, Diabetes health coach | 28 |
| JBJ | HC1_02, Diabetes health coach | 20 |
| JBJ | HC1_03, Diabetes health coach | 19 |
| JBJ | HC1_04, Diabetes health coach | 31 |
| JBJ | HC1_05, Diabetes health coach | 31 |
| JBJ | HC1_06, Diabetes health coach | 22 |
| JBJ | HC1_07, Diabetes health coach | 32 |
| JBJ | HC1_08, Diabetes health coach | 23 |
| JBJ | HC1_09, Diabetes health coach | 16 |
| JBJ | HC1_10, Diabetes health coach | 30 |
| JBJ | HC1_11, Diabetes health coach | 22 |
| JBJ | HC1_12, Diabetes health coach | 26 |
| JBJ | HC1_13, Diabetes health coach | 19 |
| JBJ | HC1_14, Diabetes health coach | 17 |
| JBJ | HC1_15, Diabetes health coach | 25 |
| JBJ | HC1_16, Diabetes health coach | 27 |

Supplement 3 – Patient-participant Interview guide

| “Can you tell me about the effect of type two diabetes on your life, starting from when you were first diagnosed?” |
| --- |
| Can you tell me about how your friends and family reacted?  (if they’ve mentioned friends or family)  Or, “what non-medical support did you have?” |
| Could you tell us how you found living with diabetes since you were diagnosed? |
| What does remission mean to you?” |
| How did you feel about trying weight loss? |
| What methods did you try? |
| How did you feel about the outcome? |
| Were there any things you tried? |

Supplement 4 – Health coach interview guide

| Could you talk a little bit about what the current client pathways are? |
| --- |
| Do you think those client pathways work well? |
| Can you tell me about how you engage participants currently? |
| Do you use any particular strategies to facilitate that engagement? |
| What do you see as the current challenges for maintaining motivation in the programme? |
| Do you foresee any barriers to setting up a new client pathway? |
